# Complete Genome Sequence of a Novel Pigeon Torque Teno Virus in China

**DOI:** 10.1128/genomeA.01076-13

**Published:** 2013-12-19

**Authors:** Zhicheng Zhang, Linlin Zhuang, Wei Dai, Huihua Mao, Dingzhen Dai

**Affiliations:** Department of Animal Science and Technology, Jinling Institute of Technology, Nanjing, Chinaa; Key Laboratory of Zoonoses of Anhui Province, Anhui Agricultural University, Hefei, Chinab

## Abstract

Torque teno virus is a nonenveloped single-stranded DNA virus infecting humans and nonprimate species. We report the complete genome sequence of a pigeon torque teno virus isolated from pigeons in Jiangsu Province, China, in 2012. This genome sequence will be useful for viral diagnostics and disease control.

## GENOME ANNOUNCEMENT

Pigeon torque teno virus (PTTV) is a novel DNA virus that was first discovered in pigeons in China. PTTV is a species-specific virus, and there is little information about how it affects different pigeons or about its molecular characteristics and evolutionary process.

Torque teno virus (TTV) is a small, nonenveloped, single-stranded, negative-sense circular DNA virus (1) belonging to the newly created family *Anelloviridae* (2). In 1997, TTV was first discovered in a Japanese patient with non-A/non-E posttransfusion hepatitis of unknown etiology, so it has been classed as a transfusion-transmitted virus in the past (3). TTV is widely distributed throughout the world, and it infects humans, nonhuman primates, tupaias, and domestic animals, including chickens, pigs, cattle, sheep, cats, and dogs (4). Analysis of genomic DNA reveals well-conserved genomic organization among the various TTV species. TTVs infect different vertebrate species, showing different genome lengths and sequence variability (5, 6). In humans, TTV infection is considered to be ubiquitous and has been detected in feces, saliva, semen, blood serum, and umbilical cord blood (7).

In this study, a total of 144 pigeon blood serum or pigeon disease samples were collected from six pigeon markets in Jiangsu Province, China. PTTV was detected in 103 out of 144 (72%) samples using specific PCR methods with the primer pair NG343 and NG344 (8). Six representative full-length PTTV genomes were amplified using reverse PCR with the primer pair PTTV1 and PTTV2.

The complete genome of PTTV is a single-stranded DNA molecule comprising 1,584 or 1,585 bp. It has two putative open reading frames (ORFs) encoding Rep replication protein (ORF V1) and Cap coat protein (ORF C1). There is a small overlapping part between the two ORFs, and translation begins in the single-stranded DNA. The ORF V1 of Jiangsu/PTTV is located at nucleotides 464 to 923 in the genome, encoding 154 amino acids, and ORF C1 was located at nucleotides 788 to 1189 in the genome, encoding 134 amino acids. However, unlike genomes of TTVs infecting mammals, the untranslated region (UTR) region of PTTV shows no significant G+C-rich region (G+C content, ~60%). Phylogenetic analyses based on the genome indicated that six Jiangsu PTTVs are closely related to one another. Sequence analysis showed that the genomes of six Jiangsu PTTVs are 96.4% to 100% homologous to that of chicken anemia virus (CAV), while they show significant difference in their genetic relationship with TTVs infecting mammals, including humans, apes, pigs, and cats.

This is believed to be the first investigation of PTTV infection in pigeons, and five complete genome sequences have been amplified. Pigeons have great commercial value worldwide; therefore, more research on the molecular characteristics and evolutionary process of PTTV is needed.

### Nucleotide sequence accession numbers.

The complete genome sequences of A/pigeons/Jiangsu/PTTV/2012 were deposited in GenBank under accession no. KF477315 to KF477319.
